# National, Regional, State, and Selected Local Area Vaccination Coverage Among Adolescents Aged 13–17 Years — United States, 2020

**DOI:** 10.15585/mmwr.mm7035a1

**Published:** 2021-09-03

**Authors:** Cassandra Pingali, David Yankey, Laurie D. Elam-Evans, Lauri E. Markowitz, Charnetta L. Williams, Benjamin Fredua, Lucy A. McNamara, Shannon Stokley, James A. Singleton

**Affiliations:** ^1^Immunization Services Division, National Center for Immunization and Respiratory Diseases, CDC; ^2^Division of Viral Diseases, National Center for Immunization and Respiratory Diseases, CDC; ^3^Leidos Health, Atlanta, Georgia; ^4^Division of Bacterial Diseases, National Center for Immunization and Respiratory Diseases, CDC.

The Advisory Committee on Immunization Practices (ACIP) recommends that adolescents aged 11–12 years routinely receive tetanus, diphtheria, and acellular pertussis (Tdap); meningococcal conjugate (MenACWY); and human papillomavirus (HPV) vaccines. Catch-up vaccination is recommended for hepatitis B (HepB); hepatitis A (HepA); measles, mumps, and rubella (MMR); and varicella (VAR) vaccines for adolescents whose childhood vaccinations are not current. Adolescents are also recommended to receive a booster dose of MenACWY vaccine at age 16 years, and shared clinical decision-making is recommended for the serogroup B meningococcal vaccine (MenB) for persons aged 16–23 years (*1*). To estimate coverage with recommended vaccines, CDC analyzed data from the 2020 National Immunization Survey–Teen (NIS-Teen) for 20,163 adolescents aged 13–17 years.* Coverage with ≥1 dose of HPV vaccine increased from 71.5% in 2019 to 75.1% in 2020. The percentage of adolescents who were up to date^†^ with HPV vaccination (HPV UTD) increased from 54.2% in 2019 to 58.6% in 2020. Coverage with ≥1 dose of Tdap, ≥1 dose (and among adolescents aged 17 years, ≥2 doses) of MenACWY remained similar to coverage in 2019 (90.1%, 89.3%, and 54.4% respectively). Coverage increased for ≥2 doses of HepA among adolescents aged 13–17 years and ≥1 dose of MenB among adolescents aged 17 years. Adolescents living below the federal poverty level^§^ had higher HPV vaccination coverage than adolescents living at or above the poverty level. Adolescents living outside a metropolitan statistical area (MSA)^¶^ had lower coverage with ≥1 MenACWY and ≥1 HPV dose, and a lower proportion being HPV UTD than adolescents in MSA principal cities. In 2020, the COVID-19 pandemic disrupted routine immunization services. Results from the 2020 NIS-Teen reflect adolescent vaccination coverage before the COVID-19 pandemic. The 2020 NIS-Teen data could be used to assess the impact of the COVID-19 pandemic on catch-up vaccination but not on routine adolescent vaccination because adolescents included in the survey were aged ≥13 years, past the age when most routine adolescent vaccines are recommended, and most vaccinations occurred before March 2020. Continued efforts to reach adolescents whose routine medical care has been affected by the COVID-19 pandemic are necessary to protect persons and communities from vaccine-preventable diseases and outbreaks.

NIS-Teen is an annual random-digit–dialed telephone survey** that monitors vaccination coverage in adolescents aged 13–17 years in all 50 states, the District of Columbia, selected local areas, and some U.S. territories.^††^ Parents or guardians of eligible adolescents are interviewed to gather sociodemographic information about the household, and consent to contact the adolescent’s vaccination provider (or providers) is requested; if permission is granted, a questionnaire is mailed to the provider (or providers) to obtain the adolescent’s vaccination history. Vaccination coverage estimates are based on provider-reported immunization records and include any vaccines administered before the 2020 NIS-Teen interview date. This report provides vaccination coverage estimates for 20,163 adolescents aged 13–17 years.^§§^ The overall household response rate^¶¶^ was 20.7%; 45.2% of adolescents with completed interviews had adequate provider data. Data were weighted and analyzed to account for the complex survey design, and T-tests using Taylor-series variance estimates were used to assess vaccination coverage differences by survey year (2020 versus 2019) and between sociodemographic groups.*** P-values <0.05 were considered statistically significant. Analyses were conducted using SAS-callable SUDAAN (version 11; RTI International). This activity was reviewed by CDC and was conducted consistent with applicable federal law and CDC policy.^†††^

## National Vaccination Coverage

In 2020, HPV vaccination coverage (≥1 dose) among adolescents was 75.1%, and 58.6% were HPV UTD (Figure) (Table 1). Coverage with ≥1 dose of Tdap and MenACWY remained high and stable (90.1% and 89.3% respectively). Among adolescents aged 17 years, coverage with ≥2 doses of MenACWY was 54.4%, similar to 2019 (53.7%); coverage increased for ≥1 dose of Men B among adolescents aged 17 years and catchup vaccination with ≥2 doses of HepA among adolescents 13−17 years from 2019. Coverage surpassed 90% for ≥2 doses of MMR, ≥3 doses of HepB, and ≥1 and ≥2 doses of varicella vaccine among adolescents without a history of varicella disease.^§§§^

**FIGURE:**
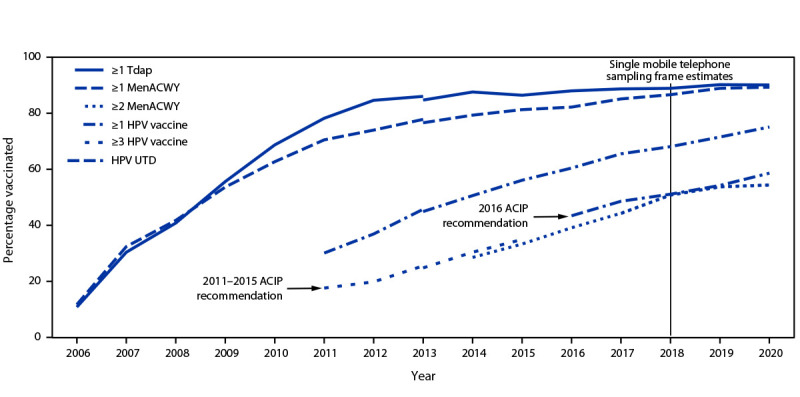
Estimated vaccination coverage with selected vaccines and doses* among adolescents aged 13–17 years, by survey year^†^ — National Immunization Survey–Teen,^§^^,^^¶^ United States, 2006–2020 **Abbreviations:** ACIP = Advisory Committee on Immunization Practices; HPV = human papillomavirus; MenACWY = quadrivalent meningococcal conjugate vaccine; NIS-teen = National Immunization Survey–Teen; Tdap = tetanus toxoid, reduced diphtheria toxoid, and acellular pertussis vaccine; UTD = up to date. * ≥1 dose Tdap at age ≥10 years; ≥1 dose MenACWY or meningococcal-unknown type vaccine; ≥2 doses MenACWY or meningococcal-unknown type vaccine, calculated only among adolescents aged 17 years at time of interview. Does not include adolescents who received their first and only dose of MenACWY at age ≥16 years; HPV vaccine, nine-valent (9vHPV), quadrivalent (4vHPV), or bivalent (2vHPV). The routine ACIP recommendation for HPV vaccination was made for females in 2006 and for males in 2011. Because HPV vaccination was recommended for males in 2011, coverage for all adolescents was not measured before that year; HPV UTD includes those with ≥3 doses, and those with 2 doses when the first HPV vaccine dose was initiated at age <15 years and at least 5 months minus 4 days elapsed between the first and second dose. ^†^ NIS-Teen implemented a revised adequate provider data definition in 2014 and retrospectively applied the revised definition to 2013 data. Estimates using a revised definition might not be directly comparable. ^§^ NIS-Teen moved in 2018 to a single-sample frame. ^¶^ ACIP revised the recommended HPV vaccination schedule in late 2016. The schedule changed from a 3-dose to 2-dose series with appropriate spacing between receipt of the first and second dose for immunocompetent adolescents initiating the series before the 15th birthday. Three doses are still recommended for adolescents initiating the series at age ≥15 years. Because of the change in definition, the graph includes estimates for ≥3 doses of HPV during 2011–2015 and the HPV UTD estimate for 2016–2020. Because HPV vaccination was recommended for males in 2011, coverage for all adolescents was not measured before that year.

**TABLE 1:** Estimated vaccination coverage with selected vaccines and doses among adolescents aged 13–17* years, by age at interview — National Immunization Survey–Teen, United States, 2020

Vaccine	Age at interview (yrs), % (95% CI)^†^					Total, % (95% CI)^†^	
	13	14	15	16	17	2020	2019
	(n = 4,276)	(n = 4,173)	(n = 3,998)	(n = 4,028)	(n = 3,688)	(N = 20,163)	(N = 18,788)
**Tdap^§^ ≥1 dose**	88.9 (87.0–90.6)	89.4 (87.1–91.3)	90.7 (88.7–92.5)	90.4 (88.3–92.1)	91.1 (88.7–93.0)	**90.1 (89.2–90.9)**	**90.2 (89.2–91.1)**
**MenACWY^¶^**							
≥1 dose	87.5 (85.3–89.4)	87.6 (85.0–89.8)	90.4 (88.6–92.0)**	89.1 (86.9–91.0)	92.3 (90.3–93.9)**	**89.3 (88.4–90.2)**	**88.9 (88.0–89.8)**
≥2 doses**^††^**	NA	NA	NA	NA	54.4 (51.2–57.5)	**54.4 (51.2–57.5)**	**53.7 (49.9–57.4)**
**HPV^§§^ vaccine**							
**All adolescents**							
≥1 dose	69.4 (66.6–72.1)	72.3 (69.4–75.0)	77.6 (75.3–79.8)**	77.2 (74.7–79.6)**	79.0 (76.4–81.4)**	**75.1 (73.9–76.2)^¶¶^**	**71.5 (70.1–72.8)**
HPV UTD***	45.6 (42.7–48.5)	56.0 (53.0–58.9)**	61.9 (58.9–64.7)**	65.5 (62.6–68.2)**	64.5 (61.5–67.4)**	**58.6 (57.3–60.0)^¶¶^**	**54.2 (52.7–55.8)**
**Females**							
≥1 dose	71.3 (67.7–74.7)	72.9 (68.4–77.0)	78.1 (74.6–81.3)**	80.3 (76.3–83.8)**	83.5 (80.8–85.9)**	**77.1 (75.4–78.7)^¶¶^**	**73.2 (71.3–75.0)**
HPV UTD	48.4 (44.3–52.5)	57.2 (52.6–61.7)**	63.7 (59.4–67.8)**	68.5 (64.0–72.6)**	70.4 (66.6–73.9)**	**61.4 (59.5–63.3)^¶¶^**	**56.8 (54.6–59.0)**
**Males**							
≥1 dose	67.5 (63.2–71.5)	71.7 (67.9–75.2)	77.1 (73.9–80.1)**	74.5 (71.1–77.6)**	74.8 (70.4–78.6)**	**73.1 (71.5–74.8)^¶¶^**	**69.8 (67.9–71.7)**
HPV UTD	42.7 (38.6–46.9)	54.8 (50.9–58.6)**	60.0 (56.1–63.9)**	62.8 (58.9–66.4)**	59.0 (54.4–63.5)**	**56.0 (54.1–57.8)^¶¶^**	**51.8 (49.7–53.9)**
**MenB ≥1 dose^†††^**	NA	NA	NA	NA	28.4 (25.5–31.5)	**28.4 (25.5–31.5)^¶¶^**	**21.8 (18.9–24.9)**
**MMR ≥2 doses**	92.5 (90.7–94.0)	92.1 (90.3–93.5)	92.5 (90.4–94.2)	93.2 (91.5–94.7)	91.6 (89.2–93.5)	**92.4 (91.6–93.2)**	**91.9 (90.8–92.8)**
**Hepatitis A vaccine ≥2 doses^§§§^**	86.5 (84.1–88.5)	84.9 (82.6–86.9)	81.5 (79.1–83.6)**	79.8 (77.5–81.8)**	77.7 (75.0–80.1)**	**82.1 (81.1–83.1)^¶¶^**	**77.1 (75.8–78.4)**
**Hepatitis B vaccine ≥3 doses**	91.8 (89.8–93.4)	93.5 (92.1–94.8)	92.5 (90.7–94.0)	93.6 (92.0–94.8)	91.4 (89.1–93.3)	**92.6 (91.8–93.3)**	**91.6 (90.6–92.6)**
**Varicella**							
History of varicella^¶¶¶^	6.8 (5.4–8.5)	6.9 (5.7–8.3)	8.7 (7.1–10.6)	7.6 (6.4–9.1)	12.0 (9.7–14.8)**	**8.4 (7.6–9.2)**	**9.1 (8.4–9.9)**
No history of varicella disease							
≥1 dose vaccine	96.2 (94.8–97.2)	95.9 (94.4–97.0)	95.3 (93.5–96.7)	95.3 (93.3–96.7)	95.2 (93.6–96.5)	**95.6 (94.9–96.2)**	**95.2 (94.3–95.9)**
≥2 doses vaccine	93.6 (92.0–95.0)	91.6 (89.6–93.2)	92.8 (90.6–94.5)	90.8 (88.3–92.9)**	90.5 (88.1–92.5)**	**91.9 (91.0–92.7)**	**90.6 (89.5–91.7)**
History of varicella or received ≥2 doses varicella vaccine	94.1 (92.6–95.3)	92.1 (90.3–93.6)	93.4 (91.4–95.0)	91.5 (89.2–93.4)	91.6 (89.5–93.4)**	**92.6 (91.7–93.3)**	**91.5 (90.4–92.4)**

**Abbreviations**: CI = confidence interval; HPV = human papillomavirus; MenACWY = quadrivalent meningococcal conjugate vaccine; MenB = serogroup B meningococcal vaccine; MMR = measles, mumps, and rubella vaccine; NA = not applicable; NIS-Teen = National Immunization Survey–Teen; Tdap = tetanus toxoid, reduced diphtheria toxoid, and acellular pertussis vaccine; UTD = up to date.

* Adolescents (20,163) in the 2020 NIS-Teen were born during January 2002–January 2008.

^†^ Estimates with 95% CI widths >20 might be unreliable.

^§^ Includes percentages receiving Tdap vaccine at age ≥10 years.

^¶^ Includes percentages receiving MenACWY or meningococcal-unknown type vaccine.

** Statistically significant difference (p<0.05) in estimated vaccination coverage by age: reference group was adolescents aged 13 years.

^††^ ≥2 doses of MenACWY or meningococcal-unknown type vaccine. Calculated only among adolescents who were aged 17 years at interview. Does not include adolescents who received 1 dose of MenACWY vaccine at age ≥16 years.

^§§^ HPV vaccine, nine-valent (9vHPV), quadrivalent (4vHPV), or bivalent (2vHPV). For ≥1 HPV dose measure and HPV UTD measure, percentages are reported among females and males combined (20,163) and among females only (9,576) and among males only (10,587).

^¶¶^ Statistically significant difference (p<0.05) compared with 2019 NIS-Teen estimates.

*** HPV UTD includes those with ≥3 doses, and those with 2 doses when the first HPV vaccine dose was initiated before age 15 years and there was at least 5 months minus 4 days between the first and second dose. This update to the HPV recommendation occurred in December 2016.

^†††^ ≥1 dose of MenB. Calculated only among adolescents who were aged 17 years at interview. Administered based on individual clinical decision.

^§§§^ In July 2020, ACIP revised recommendations for HepA vaccination to include catch-up vaccination for children and adolescents aged 2–18 years who have not previously received HepA vaccine at any age. https://www.cdc.gov/mmwr/volumes/69/rr/rr6905a1.htm?s_cid=rr6905a1

^¶¶¶^ By parent or guardian report or provider records.

## Vaccination Coverage by Selected Characteristics

Among adolescents living in non-MSA areas, vaccination coverage was lower compared with those living in MSA principal cities with ≥1 dose MenACWY (85.7% versus 90.2% [−4.5 percentage points]), ≥1 dose HPV (68.0% versus 77.8% [−9.8 percentage points]), and ≥2 doses HepA (76.2% versus 83.6% [−7.4 percentage points])], and being HPV UTD (49.2% versus 60.4% [−11.2 percentage points]) (Table 2). These MSA disparities persisted among adolescents at or above the poverty level but were not significant among those below the poverty level for HPV UTD status and ≥2 dose–HepA coverage. The coverage disparity in non-MSA areas compared with MSA principal cities among adolescents living at or above the poverty level were largest for HPV UTD status (46.0% versus 59.8% [−13.8 percentage points]), ≥1-dose HPV coverage (64.9% versus 76.2% [−11.3 percentage points], and ≥2-dose HepA coverage (74.4% versus 83.6% [−9.2 percentage points]). Coverage varied by jurisdiction (Supplementary Table, https://stacks.cdc.gov/view/cdc/109214), race and ethnicity,^¶¶¶^ and health insurance status.****

**TABLE 2:** Estimated vaccination coverage with selected vaccines and doses among adolescents aged 13–17* years, by metropolitan statistical area† and by poverty level — National Immunization Survey–Teen, United States, 2020

Vaccine	MSA, % (95% CI)^§^			Below poverty level, % (95% CI)^§^			At or above poverty level, % (95% CI)^§^		
	Non-MSA	MSA nonprincipal city	MSA principal city	Non-MSA	MSA nonprincipal city	MSA principal city	Non-MSA	MSA nonprincipal city	MSA principal city
	(n = 3,678)	(n = 8,409)	(n = 8,076)	(n = 631)	(n = 865)	(n = 1,352)	(n = 2,938)	(n = 7,246)	(n = 6,420)
**Tdap^¶^ ≥1 dose**	90.7 (88.7–92.3)	90.6 (89.3–91.8)	89.3 (87.7–90.7)	93.1 (89.7–95.5)	89.0 (84.7–92.1)	89.6 (86.3–92.2)	89.8 (87.4–91.9)	91.1 (89.7–92.3)	89.2 (87.3–90.9)
MenACWY**									
≥1 dose	85.7 (83.7–87.5)^††^	89.4 (87.9–90.7)	90.2 (88.7–91.5)	86.1 (81.8–89.5)^††^	87.2 (82.6–90.6)	91.6 (88.8–93.7)	85.6 (83.2–87.7)^††^	90.2 (88.6–91.5)	89.4 (87.5–91.0)
≥2 doses^§§^	50.1 (43.4–56.9)	58.5 (54.0–62.8)^††^	50.6 (45.2–56.1)	47.4 (33.5–61.7)	47.6 (33.0–62.7)	48.6 (35.8–61.7)	50.2 (42.3–58.0)	61.2 (56.6–65.6)^††^	50.2 (44.1–56.4)
**HPV^¶¶^ vaccine**									
**All adolescents**									
≥1 dose	68.0 (65.3–70.6)^††^	74.2 (72.5–75.9)^††^	77.8 (75.8–79.6)	73.6 (67.8–78.7)^††^	83.6 (79.5–87.0)	85.7 (82.0–88.7)	64.9 (61.7–67.9)^††^	73.1 (71.3–74.9)^††^	76.2 (74.0–78.3)
HPV UTD***	49.2 (46.3–52.1)^††^	59.1 (57.2–61.0)	60.4 (58.2–62.6)	56.7 (50.3–62.9)	63.8 (58.1–69.2)	64.4 (59.2–69.3)	46.0 (42.9–49.3)^††^	58.4 (56.4–60.4)	59.8 (57.4–62.2)
**Females**									
≥1 dose	67.8 (63.7–71.7) ^††^	76.7 (74.5–78.8)	79.8 (76.9–82.4)	75.2 (66.4–82.2)^††^	84.4 (78.9–88.6)	87.2 (82.0–91.0)	63.6 (58.6–68.2)^††^	75.7 (73.3–78.0)	78.8 (75.7–81.7)
HPV UTD	50.3 (46.0–54.6)^††^	62.2 (59.6–64.7)	63.2 (59.9–66.4)	56.9 (47.6–65.8)	65.3 (56.8–72.9)	66.0 (58.2–73.0)	46.8 (42.0–51.6)^††^	61.9 (59.1–64.5)	63.5 (60.0–67.0)
**Males**									
≥1 dose	68.1 (64.6–71.5)^††^	71.9 (69.3–74.4)^††^	75.8 (73.2–78.3)	71.6 (63.7–78.4)^††^	82.9 (76.5–87.8)	84.3 (78.8–88.6)	66.1 (61.9–70.0)^††^	70.7 (68.0–73.3)	73.7 (70.5–76.6)
HPV UTD	48.1 (44.3–52.0)^††^	56.2 (53.4–58.9)	57.8 (54.8–60.7)	56.4 (47.8–64.7)	62.5 (54.5–69.8)	62.9 (55.8–69.4)	45.4 (41.2–49.7)^††^	55.2 (52.2–58.1)	56.2 (52.8–59.5)
**MMR ≥2 doses**	92.8 (91.0–94.2)	92.4 (91.2–93.5)	92.3 (90.9–93.5)	93.6 (89.4–96.2)	89.5 (83.8–93.3)	90.8 (86.2–94.0)	92.3 (90.2–94.0)	92.9 (91.6–93.9)	92.4 (90.9–93.6)
**Hepatitis A vaccine ≥2 doses^†††^**	76.2 (73.7–78.5)^††^	82.0 (80.6–83.4)	83.6 (81.9–85.2)	80.4 (75.0–84.9)	82.1 (77.1–86.3)	83.0 (78.1–87.0)	74.4 (71.5–77.0)^††^	81.8 (80.2–83.3)	83.6 (81.7–85.3)
**Hepatitis B vaccine ≥3 doses**	92.4 (90.6–93.9)	92.9 (91.7–93.9)	92.3 (90.9–93.5)	92.8 (89.0–95.4)	91.0 (86.3–94.2)	89.9 (85.5–93.2)	92.0 (89.8–93.8)	93.1 (91.9–94.1)	92.9 (91.5–94.1)
**Varicella**									
History of varicella^§§§^	10.1 (8.6–11.8)	8.2 (7.2–9.4)	8.0 (6.9–9.4)	9.8 (6.9–13.6)	13.3 (9.4–18.5)	9.3 (6.4–13.3)	10.3 (8.6–12.3)^††^	7.2 (6.2–8.4)	7.8 (6.5–9.3)
**No history of varicella disease**									
≥1 dose vaccine	96.1 (94.6–97.1)	95.8 (94.8–96.6)	95.3 (94.1–96.2)	95.9 (91.9–97.9)	96.9 (94.6–98.2)	94.0 (90.0–96.4)	96.1 (94.4–97.3)	95.5 (94.3–96.4)	95.5 (94.2–96.5)
≥2 doses vaccine	92.5 (90.7–94.0)	92.0 (90.6–93.2)	91.6 (90.1–92.9)	94.2 (90.1–96.6)	87.9 (81.2–92.4)	90.7 (85.7–94.0)	91.7 (89.4–93.5)	92.4 (91.0–93.5)	91.6 (90.0–93.0)
History of varicella or received ≥2 doses VAR	93.2 (91.6–94.6)	92.7 (91.4–93.8)	92.3 (90.9–93.5)	94.7 (91.0–97.0)	89.5 (83.6–93.4)	91.5 (87.0–94.6)	92.5 (90.5–94.1)	92.9 (91.6–94.0)	92.2 (90.7–93.5)

**Abbreviations**: CI = confidence interval; HPV = human papillomavirus; MenACWY = quadrivalent meningococcal conjugate vaccine; MMR = measles, mumps, and rubella vaccine; MSA = metropolitan statistical area; NIS-Teen = National Immunization Survey–Teen; Tdap = tetanus toxoid, reduced diphtheria toxoid, and acellular pertussis vaccine; UTD = up to date; VAR = varicella vaccine.

* Adolescents (20,163) in the 2020 NIS-Teen were born during January 2002–January 2008.

^†^ MSA status was determined based on household-reported county of residence and was grouped into three categories: MSA principal city, MSA nonprincipal city, and non-MSA. MSA and principal city were as defined by the U.S. Census Bureau https://www.census.gov/programs-surveys/metro-micro.html). Non-MSA areas include urban populations not located within an MSA as well as completely rural areas.

^§^ Estimates with 95% CI widths >20 might not be reliable.

^¶^ Includes percentages receiving Tdap vaccine at age ≥10 years.

** Includes percentages receiving MenACWY and meningococcal-unknown type vaccine.

^††^ Statistically significant difference (p<0.05) in estimated vaccination coverage by metropolitan statistical area; referent group was adolescents living in MSA principal city areas.

^§§^ ≥2 doses of MenACWY or meningococcal-unknown type vaccine. Calculated only among adolescents who were aged 17 years at interview. Does not include adolescents who received 1 dose of MenACWY vaccine at age ≥16 years.

^¶¶^ HPV vaccine, nine-valent (9vHPV), quadrivalent (4vHPV), or bivalent (2vHPV) in females and males combined.

*** HPV UTD includes those with ≥3 doses, and those with 2 doses when the first HPV vaccine dose was initiated before age 15 years and there was at least 5 months minus 4 days between the first and second dose. This update to the HPV recommendation occurred in December 2016.

^†††^ In July 2020, ACIP revised recommendations for HepA vaccination to include catch-up vaccination for children and adolescents aged 2–18 years who have not previously received HepA vaccine at any age. https://www.cdc.gov/mmwr/volumes/69/rr/rr6905a1.htm?s_cid=rr6905a1

^§§§^ By parent or guardian report or provider records.

## COVID-19 Pandemic Effects on HPV Vaccination

The COVID-19 pandemic was declared a national emergency on March 13, 2020. To evaluate the impact of the pandemic on HPV vaccination, CDC conducted two analyses comparing the 2019 and 2020 NIS-Teen samples. Historically, HPV vaccination coverage has been lower than coverage with most other routine vaccines, allowing for more catch-up vaccinations among adolescents aged 13–17 years. Most adolescents had initiated HPV vaccination before March 1 in both survey years (69.1% in 2019 and 73.6% in 2020). An additional 2.4% and 1.5% of adolescents initiated the series after this date in 2019 and 2020, respectively.

The second analysis evaluated adolescents in the 2020 NIS-Teen sample who had not received HPV vaccine before March 1 and whose parent or guardian was interviewed on or after that date. This cohort was compared with a similarly constructed cohort using 2019 NIS-Teen data. Cumulative daily HPV vaccination initiation estimates from March through December for these cohorts were calculated using the Kaplan-Meier method.^††††^ Among the 4,918 adolescents who had not received HPV vaccine as of March 1, 2019 (26.2% of the total sample), 452 (15.0%) initiated the series by mid-December 2019. Among the 4,527 adolescents who had not received HPV vaccine as of March 1, 2020, (22.5% of total sample), 282 (15.2%) initiated the series by mid-December 2020. HPV vaccination initiation in the 2020 cohort was lower than that in the 2019 cohort by April. The difference between the two cohorts was largest in August and September (4.9 percentage points lower in 2020 in both months) but narrowed in subsequent months and was no longer significant by end of November (Supplementary Figure, https://stacks.cdc.gov/view/cdc/109215).

## Discussion

NIS-Teen 2020 data indicate that although ≥1 dose HPV coverage and HPV UTD status continue to increase, they remain lower than coverage with most other routinely recommended vaccines. Improvements in HPV vaccination coverage are crucial to lowering rates of HPV-attributable cancers in the United States. Coverage with ≥1 dose of Tdap and MenACWY vaccines remains high and stable, while coverage with ≥2 doses of MenACWY remains low, indicating the need for increased awareness of the importance of the booster dose.

Disparities in vaccination coverage by MSA and poverty level persist. Among adolescents living at or above the poverty level, those in non-MSAs had lower HPV UTD status and coverage with ≥2 doses of HepA than adolescents in MSA principal cities. Further investigation is needed to understand this disparity and more generally, the relationship between socioeconomic level, geographic location, barriers to vaccination such as vaccination access, and vaccine confidence. Persons living below the poverty level might have better access to the VFC program,^§§§§^ which provides vaccines to children whose parents or guardians otherwise might not be able to afford them. Adolescents living below the poverty level have previously been shown to have higher HPV vaccine coverage (*2*–*4*).

Although HPV vaccination continues to increase in the United States, and coverage for most other routine vaccinations remains high and stable, the COVID-19 pandemic threatens these achievements. An analysis of immunization information systems data from 10 U.S. jurisdictions during March–May 2020 compared with the same period in 2018 and 2019 identified a substantial decrease in the number of vaccine doses administered to children and adolescents in 2020. Increases in doses administered were noted during June–September 2020 but did not appear sufficient to offset the decline during March–May 2020 (*5*). Analysis of adolescents in the 2019 and 2020 NIS-Teen data who were aged ≥13 years and had not initiated HPV vaccination as of March 1 showed lower series initiation initially from April through the end of October in 2020 compared with 2019; however, initiation of the HPV series in 2019 and 2020 was similar by November–December. Although this is encouraging, the NIS-Teen data cannot yet be used to assess the potential impact of the pandemic on adolescents who were due to receive vaccinations at age 11–12 years. As adolescents aged 11–12 years who were due to receive routine vaccinations during the pandemic age into the NIS-Teen survey sample (13–17 years), the full impact of the COVID-19 pandemic can be assessed.

The findings in this report are subject to at least three limitations. First, the household response rate was 20.7%, and 45.2% of respondents had adequate provider data. Low survey response rates can increase potential biases if survey participants differ from nonrespondents (*6*). Second, bias in estimates might remain after adjustment for household and provider nonresponse and phoneless households. A recent survey error assessment indicated that NIS-Teen estimates might underestimate true coverage, with the largest underestimation for Tdap vaccine (−5.3 percentage points).^¶¶¶¶^ Little evidence exists of a change in survey accuracy between 2019 and 2020.***** Finally, opportunity is limited to assess the effect of the pandemic on routine coverage using 2020 NIS-Teen data; because many vaccines are recommended for children aged 11–12 years, most adolescents aged 13–17 years received their routine vaccinations before the pandemic started.

Health care providers should review patient vaccination records and administer any vaccines or doses that are due. Children and adolescents aged 12–17 years are also eligible (those aged 16–17 years as of December 11, 2020 and those aged 12–15 years as of May 10, 2021) for a COVID-19 vaccine, which may be administered with other vaccines at the same visit (*7*). Ensuring that routine vaccination is maintained and that adolescents catch up on any missed doses is essential to protecting persons and communities from vaccine-preventable diseases and outbreaks.

**Summary**

**What is already known about this topic?**

Tetanus, diphtheria, and acellular pertussis (Tdap), meningococcal conjugate (MenACWY), and human papillomavirus (HPV) vaccines are routinely recommended for adolescents.

**What is added by this report?**

In 2020, adolescent coverage with Tdap and the first dose of MenACWY remained high and continued to improve for HPV vaccines, with some disparities. Adolescents living outside a metropolitan statistical area (MSA) had lower vaccination coverage compared with adolescents living in MSA principal cities.

**What are the implications for public health?**

Results from the 2020 National Immunization Survey–Teen reflect adolescent vaccination coverage before the COVID-19 pandemic. Efforts to reach adolescents whose routine medical care has been affected by the pandemic are necessary to protect adolescents and communities from vaccine-preventable diseases and outbreaks.

## Footnotes

* Eligible participants were born during January 2002–January 2008. Tetanus toxoid, reduced diphtheria toxoid, and acellular pertussis vaccine represents coverage with ≥1 Tdap dose at age ≥10 years. Meningococcal conjugate vaccine represents coverage with the quadrivalent meningococcal conjugate vaccine or meningococcal-unknown type vaccine. HPV vaccination coverage includes receipt of any HPV vaccine and does not distinguish between nine-valent (9vHPV), quadrivalent (4vHPV), or bivalent (2vHPV) vaccines. Some adolescents might have received more than the 2 or 3 recommended HPV vaccine doses. Hepatitis A, hepatitis B, varicella, and measles, mumps, and rubella vaccines are considered childhood vaccinations and are recommended for adolescents who are not up to date with these vaccinations. Estimates in this report include those who might have received vaccinations on-time or as catch-up. Except as noted, coverage estimates for ≥1 and ≥2 varicella vaccine doses were obtained among adolescents with no history of varicella disease. Influenza vaccination coverage data are not included in this report but are available at https://www.cdc.gov/flu/fluvaxview/index.htm.

† Adolescents were considered to be up to date with HPV vaccination if they had received ≥3 doses, or if each of the following applied: 1) they had received 2 doses; 2) the first dose was received before their 15th birthday; and 3) the difference between dates of first and second doses was ≥5 months minus 4 days, the absolute minimum interval between the first and second doses. https://www.cdc.gov/vaccines/programs/iis/cdsi.html

§ Adolescents were classified as being below the federal poverty level if their total family income was less than the level specified for the applicable family size and number of children aged <18 years. All others were classified as at or above the poverty level (https://www.census.gov/data/tables/time-series/demo/income-poverty/historical-poverty-thresholds.html). Poverty status was unknown for 711 adolescents.

¶ MSA status was determined from household reported city and county of residence and was grouped into three categories: MSA principal city, MSA nonprincipal city, and non-MSA. MSA and MSA principal city were as defined by the U.S. Census Bureau (https://www.census.gov/programs-surveys/metro-micro.html). Non-MSA areas include urban populations not located within an MSA and completely rural areas.

** Persons living in all identified mobile-telephone households were eligible for interview. Sampling weights were adjusted for single frame (mobile telephone), nonresponse, noncoverage, and overlapping samples of mixed telephone users. A description of NIS-Teen single-frame survey methodology and its effect on reported vaccination estimates is available at https://www.cdc.gov/vaccines/imz-managers/coverage/teenvaxview/pubs-presentations/dual-to-single-frame-teen.html.

†† Local areas that received federal immunization funds under Section 317 of the Public Health Service Act were sampled separately. Those included Chicago, Illinois; New York, New York; Philadelphia County, Pennsylvania; Bexar County, Texas; and Houston, Texas. Two territories were sampled separately in 2020: Guam and Puerto Rico.

§§ The 2020 NIS-Teen sample included 9,576 females and 10,587 males. Adolescents from Guam (300), and Puerto Rico (169) were excluded from the national estimates.

¶¶ The Council of American Survey Research Organizations response rate is the product of three other rates: 1) the resolution rate (the proportion of telephone numbers that can be identified as either for business or residence), 2) the screening rate (the proportion of qualified households that complete the screening process), and 3) the cooperation rate (the proportion of contacted eligible households for which a completed interview is obtained).

*** The NIS-Teen methodology for weighting and synthesizing provider-reported vaccination histories has been previously described. https://www.cdc.gov/vaccines/imz-managers/nis/downloads/NIS-TEEN-PUF19-DUG.pdf

††† 45 C.F.R. part 46.102(l)(2); 21 C.F.R. part 56; 42 U.S.C. Sect. 241(d); 5 U.S.C. Sect. 552a; 44 U.S.C. Sect. 3501 et seq.

§§§ HepA, HepB, VAR, and MMR vaccines are considered childhood vaccinations and are recommended for adolescents who are not up to date with these vaccinations. Estimates in this report include those who might have received vaccinations on-time or as catch-up.

¶¶¶ Hispanic adolescents had lower coverage for ≥1 Tdap (−3.9 percentage points), ≥2 MMR (−4.0 percentage points), ≥3 HepB (−4.3 percentage points), ≥1 Var (−2.6 percentage points), and ≥2 Var (−3.2 percentage points) than White adolescents. Black adolescents had lower coverage for ≥2 MenACWY (−2.5 percentage points) than White adolescents. Results showed higher HPV vaccine coverage (≥1 dose) for Black, Hispanic, American Indian or Alaska Native, Asian, and multiracial adolescents than White adolescents. Results also showed higher HPV UTD for Black, Hispanic, and multiracial adolescents. The higher HPV coverage has been observed for Black and Hispanic adolescents compared with White adolescents for several years. https://www.cdc.gov/vaccines/imz-managers/coverage/teenvaxview/pubs-presentations/NIS-teen-vac-coverage-estimates-2020-tables.html#table-01

**** Adolescents with any Medicaid insurance had lower coverage for ≥1 Tdap (−3.1 percentage points) but higher coverage with ≥1 HPV (6.1 percentage points) compared with adolescents with private health insurance. Adolescents who were uninsured had lower coverage for all routine vaccinations (Tdap, MenACWY, and HPV vaccines) compared with adolescents with private health insurance. https://www.cdc.gov/vaccines/imz-managers/coverage/teenvaxview/pubs-presentations/NIS-teen-vac-coverage-estimates-2020-tables.html#table-02

†††† Kaplan-Meier methods were used to calculate cumulative daily vaccination estimates from March–December 2019 and from March–December 2020. In 2019, 4,918 adolescents had not received a dose of HPV vaccine and had not been interviewed as of March 1, 2019; 452 were vaccinated between March–December 2019. In 2020, 4,527 adolescents had not received a dose of HPV vaccine and had not been interviewed as of March 1, 2020; 282 were vaccinated between March–December 2020.

§§§§ Children aged ≤18 years who are Medicaid-eligible, uninsured, or American Indian or Alaska Native (as defined by the Indian Health Care Improvement Act) are eligible to receive vaccines from providers through the Vaccines for Children (VFC) program. Children categorized as “underinsured” because their health plans do not include coverage for recommended vaccinations are eligible to receive VFC vaccines if they are served by a rural health clinic or federally qualified health center or under an approved deputization agreement. https://www.cdc.gov/vaccines/programs/vfc/providers/eligibility.html

¶¶¶¶ An assessment of validity of the 2019 NIS-Teen estimates has been reported (https://www.cdc.gov/vaccines/imz-managers/nis/downloads/NIS-TEEN-PUF19-DUG.pdf). NIS-Teen vaccination coverage estimates tended to be slightly low compared with true values derived after adjusting for noncoverage, nonresponse, and vaccination underascertainment, reaching up to 5.3 percentage points too low for Tdap. This was primarily attributed to underascertainment of vaccinations by the NIS provider record check. The validity of estimates did not change from 2018 to 2019.

*****
https://www.cdc.gov/vaccines/imz-managers/coverage/teenvaxview/pubs-presentations/NIS-teen-vac-coverage-estimates-2020-tables.html#table-03
